# Implementation strategies: recommendations for specifying and reporting

**DOI:** 10.1186/1748-5908-8-139

**Published:** 2013-12-01

**Authors:** Enola K Proctor, Byron J Powell, J Curtis McMillen

**Affiliations:** 1 George Warren Brown School of Social Work, Washington University in St. Louis, One Brookings Drive, Campus Box 1196, St. Louis, MO, USA; 2 School of Social Service Administration, The University of Chicago, 969 E. 60th Street, Chicago, IL, USA

**Keywords:** Implementation strategies, Implementation research, Measurement, Methodology

## Abstract

Implementation strategies have unparalleled importance in implementation science, as they constitute the ‘how to’ component of changing healthcare practice. Yet, implementation researchers and other stakeholders are not able to fully utilize the findings of studies focusing on implementation strategies because they are often inconsistently labelled and poorly described, are rarely justified theoretically, lack operational definitions or manuals to guide their use, and are part of ‘packaged’ approaches whose specific elements are poorly understood. We address the challenges of specifying and reporting implementation strategies encountered by researchers who design, conduct, and report research on implementation strategies. Specifically, we propose guidelines for naming, defining, and operationalizing implementation strategies in terms of seven dimensions: actor, the action, action targets, temporality, dose, implementation outcomes addressed, and theoretical justification. Ultimately, implementation strategies cannot be used in practice or tested in research without a full description of their components and how they should be used. As with all intervention research, their descriptions must be precise enough to enable measurement and ‘reproducibility.’ We propose these recommendations to improve the reporting of implementation strategies in research studies and to stimulate further identification of elements pertinent to implementation strategies that should be included in reporting guidelines for implementation strategies.

## The need for better specification and reporting of implementation strategies

Implementation strategies have unparalleled importance in implementation science, as they constitute the ‘how to’ component of changing healthcare practice. Comprising the specific means or methods for adopting and sustaining interventions [1], implementation strategies are recognized as necessary for realizing the public health benefits of evidence-based care [2]. Accordingly, developing strategies to overcome barriers and increase the pace and effectiveness of implementation is a high research priority [3-7].

While the evidence for particular implementation strategies is increasing [8], limitations in their specification pose serious problems that thwart their testing and hence the development of an evidence-base for their efficiency, cost, and effectiveness. Implementation strategies are often inconsistently labelled and poorly described [9], are rarely justified theoretically [10,11], lack operational definitions or manuals to guide their use, and are part of ‘packaged’ approaches whose specific elements are poorly understood [12]. The literature on implementation has been characterized as a ‘Tower of Babel’ [13], which makes it difficult to search for empirical studies of implementation strategies, and to compare the effects of different implementation strategies through meta-analyses [9]. Worse yet, the lack of clarity and depth in the description of implementation strategies within the published literature precludes replication in both research and practice. As with all intervention research, implementation strategies need to be fully and precisely described, in detail sufficient to enable measurement and ‘reproducibility’ [14] of their components.

The purpose of this article is to provide guidance to researchers who are designing, conducting, and reporting studies by proposing specific standards for characterizing implementation strategies in sufficient detail. We begin by providing a brief introduction to implementation strategies, including how the broad term has been defined as well as some examples of implementation strategies. Thereafter we suggest an extension of existing reporting guidelines that provides direction to researchers with regard to naming, clearly describing, and operationalizing implementation strategies.

### Definitions and examples of implementation strategies

Implementation strategies can be defined as methods or techniques used to enhance the adoption, implementation, and sustainability of a clinical program or practice [15]. A growing literature on implementation strategies provides a window into their type, range, and nature. They include ‘top down/bottom up,’ ‘push/pull,’ and ‘carrot/stick’ tactics, and typically involve ‘package’ approaches [16]. They include methods for provider training and decision support; intervention-specific tool kits, checklists, and algorithms; formal practice protocols and guidelines; learning collaboratives, business strategies and organizational interventions from management science (*e.g.*, plan-do-study-act cycles [17] and ‘lean thinking’ [18]); and economic, fiscal, and regulatory strategies.

The complexity of implementation strategies can vary widely. For instance, some implementation efforts may involve a single component strategy, such as disseminating treatment guidelines in the hopes of changing clinicians’ behavior (*e.g.*, Azocar *et al.*[19]). These strategies have been referred to as discrete strategies in the literature [20,21], though they have also been called ‘implementation interventions’ [22], ‘actions’ [23], and ‘specified activities’ [23]. A number of publications provide lists and taxonomies that attempt to reflect the range of these strategies [20,24-26]. For example, Powell *et al.*[20] compiled a ‘menu’ of 68 implementation strategies, grouped by six key processes: planning (*e.g.*, conducting a local needs assessment, developing a formal implementation plan), educating (*e.g.*, conduct educational meetings, distribute educational materials), financing (*e.g.*, alter incentive/allowance structures, access new funding), restructuring (*e.g.*, revise professional roles), managing quality (*e.g.*, provide clinical supervision, audit and feedback, reminders), and attending to policy context (*e.g.*, creating or changing credentialing and/or licensure requirements) [20]. Michie *et al.*[26] focused on a more granular level in their published taxonomy of 93 behavior change techniques (*e.g.*, punishment, prompts/cues, material reward, habit formation, etc.), many of which could be used to further specify implementation strategies as well.

Most often a number of strategies are combined to form a multifaceted strategy such as training, consultation, and audit and feedback. There are also a number of manualized and branded multifaceted implementation strategies, such as the ‘ARC’ organizational implementation strategy [27,28], the Institute for Healthcare Improvement’s learning collaborative [29] and framework for spread models [30], the Getting to Outcomes framework [31], and the Replicating Effective Programs (REP) framework [32,33]. The REP framework for instance, includes a number of discrete or component implementation strategies across four phases: pre-conditions (*e.g.*, identifying need, identifying barriers), pre-implementation (*e.g.*, developing a community working group), implementation (*e.g.*, training, technical assistance, feedback and refinement), and maintenance and evolution (*e.g.*, re-customize delivery as need arises) [33]. Some authors have simply used the term ‘implementation strategy’ to refer to these multi-faceted implementation strategies comprised of multiple ‘implementation interventions’ [15], whereas others have referred to ‘implementation programs’ to be inclusive of all of the component implementation strategies utilized in an implementation effort [34].

We have chosen to use the term ‘implementation strategy’ to be inclusive of both single component and multi-faceted implementation strategies, and we purposefully attempt to avoid the word ‘intervention’ largely to reduce the chance that clinical interventions and implementation interventions be confused [23]. That said, we acknowledge that some interventions can be used as either implementation strategies or interventions in their own right. For instance, the ‘ARC’ intervention [28,35] was designed as an organizational improvement strategy (*i.e.*, not necessarily as a method of implementing other clinical interventions). A randomized trial of ARC as a ‘standalone’ intervention has shown it to be effective in improving organizational culture, climate, and work attitudes as well as clinical outcomes for youth [27,36]. However, it also has been used as a strategy to implement a psychosocial intervention (Multisystemic Therapy) [28]. In cases where a strategy may be conceptualized as an improvement intervention in its own right (*i.e.*, independent of the clinical intervention being implemented) it may be useful to employ a 2 x 2 factorial design, in which both the implementation strategy and the clinical intervention are compared independently and in combination to a no treatment control. The complexity of even making the distinction between an implementation strategy and an independent intervention highlights the importance of carefully specifying the strategy in the manner that we describe below, so as to ensure that consumers of the resulting research understand how, when, why, and where the strategy is likely to be effective.

As evidenced by many of the examples above, interest has been high and progress has been made in the identification, development, and testing of implementation strategies. However, definitions and descriptions of implementation strategies in the literature often lack the clarity required to interpret study results and build upon the knowledge gained through the replication and extension of the research. This signals the need for more guidance that would assist researchers designing, conducting, and reporting implementation studies.

### Prerequisites to studying implementation strategies empirically

The study of implementation strategies should be approached in a similar fashion as evidence-based interventions (EBIs), for strategies are in fact a type of intervention. Accordingly, their specification carries the same demands as treatment specification: If they are to be scientifically tested, communicated clearly in the literature, and accurately employed in actual healthcare practice, they must be specified both conceptually and operationally [37]. There are a number of prerequisites to the measurement of implementation strategies, many of which are detailed below. They are also listed in Table 1, along with examples, resources, or tools from the literature (when available) for advancing the state of measurement.

**Table 1:** Prerequisites to measuring implementation strategies

**Prerequisite**	**Requirements**	**Resource(s) & example(s)**
**1) Name it**	Name the strategy, preferably using language that is consistent with existing literature.	Cochrane EPOC [25]
		Mazza *et al.*[24]
		Powell *et al.*[20]
**2) Define it**	Define the implementation strategy and any discrete components operationally	Abraham & Michie [38]
		Powell *et al.*[20]
		Michie *et al.*[26]
**3) Specify it**		
a) The actor	Identify who enacts the strategy (*e.g.*, administrators, payers, providers, patients/consumers, advocates, etc.).	Kauth *et al.*[39] describe the characteristics, qualifications, and roles of an external facilitator
b) The action	Use active verb statements to specify the specific actions, steps, or processes that need to be enacted.	Rapp *et al.*’s [40] operational definition of ‘leadership’
c) Action target	Specify targets according to conceptual models of implementation	Tabak *et al.*[41]
		Damschroder *et al.*[42]
	Identify unit of analysis for measuring implementation outcomes	
		Flottorp *et al.*[43]
		Cane *et al.*[44]
		Michie *et al.*[45]
		Landsverk *et al.*[46]
		Proctor *et al.*[47]
d) Temporality	Specify when the strategy is used	Magnabosco [21]
		Chinman *et al.*[31]
		Kilbourne *et al.*[33]
e) Dose	Specify dosage of implementation strategy	Atkins *et al.*[48] recorded frequency of support received by opinion leaders
f) Implementation outcome affected	Identify and measure the implementation outcome(s) likely to be affected by each strategy	Proctor *et al.*[47]
		Proctor & Brownson [49]
		Proctor *et al.*[50]
g) Justification	Provide empirical, theoretical, or pragmatic justification for the choice of implementation strategies.	Theoretical:
		Eccles *et al.*[51]
		Grol *et al.*[52]
		Empirical:
		Cochrane EPOC [53]
		Grimshaw *et al.*[8]
		Pragmatic:
		Oxman *et al.*[54]
		Wensing *et al.*[55] suggest brainstorming as a low-cost, low intensity method of linking strategies to identified barriers

The complexity of implementation strategies poses one of the greatest challenges to their clear description, operational definition, and measurement. Implementation strategies are inherently complex social interventions, as they address multifaceted and complicated processes within interpersonal, organizational, and community contexts [12,56-58]. Implementation strategies must be capable of dealing with the contingencies of various service systems, sectors, of care, and practice settings, as well as the human capital challenge of staff training and support. They must tackle a myriad of barriers to evidence-based care [59,60] and the various properties of interventions that make them more or less amenable to implementation [52]. All these factors significantly contribute to the challenge of measuring, testing, and effectively employing implementation strategies in actual healthcare practice. We attempt to provide this guidance by discussing fundamental principles for naming, defining, and specifying implementation strategies, all of which are prerequisites to studying them empirically.

1. **Name it**

To be measured, an implementation strategy must first be named or labelled. While this may seem simplistic or self-evident, Gerring [61] draws our attention to three problems that ‘…plague the social science lexicon: homonymy (multiple meanings for the same term), synonymy (different terms with the same, or overlapping, meanings), and instability (unpredictable changes in the foregoing).’ Certainly, these problems are evident within the dissemination and implementation science literature [13,62-64], and this makes it difficult to search the empirical literature, conduct meta-analyses, and ultimately, to build a body of evidence that supports the use of specific strategies in particular contexts [9,64]. For example, Brouwers *et al.*[2] found their review of studies of implementation strategies for cancer screening programs hampered by the inconsistent labelling of strategies and other specification issues related to the description and justification of selected strategies.

Given the confusion caused by poorly labelled implementation strategies and the call for the harmonization of terminology, constructs, and measures in implementation science [62], implementation stakeholders should be thoughtful as they name implementation strategies, preferably drawing upon the same terms as other researchers in the field when possible. A number of sources that have compiled implementation strategies may be helpful in identifying potentially appropriate names [20,24,25]. When different terms are used (or created), they should be carefully distinguished from strategies that are already more established in the literature. It should be noted that naming may be more complicated with multifaceted and blended strategies [20] that contain a wide variety of discrete implementation strategies. In these cases, every effort should be made to specify the discrete or component parts of the implementation strategy. For example, Forsner *et al.*[65] described a number of components to a multifaceted implementation strategy to support the implementation of clinical guidelines, including the formation of local implementation teams, the development of implementation plans, documentation of quality indicators, academic outreach detailing, etc.

2. **Define it**

A second step is to define the implementation strategy conceptually. This is distinct from the operationalization of the strategy, which will be addressed below. For example, audit and feedback can be defined conceptually as ‘any summary of clinical performance of health care over a specified period of time’ that can be provided in a written, electronic, or verbal format [66]. A conceptual definition gives a general sense of what the strategy may involve, and allows the reader to more fully discern whether or not the current usage is consistent with other uses of the term represented in the literature. Defining more complex multifaceted and/or blended implementation strategies also requires that each of the discrete strategies or components are distinguished and conceptually defined. Many of the existing taxonomies [20,24,25] provide conceptual definitions that can prove helpful in generating a better understanding of implementation strategies. Indeed, both naming and defining implementation strategies conceptually makes it possible to distinguish one strategy from another. Yet this is not sufficient for full specification. For instance, while the strategy audit and feedback may have a commonly recognized name and definition, it can be delivered in a multitude of ways in actual practice. Eccles *et al.*[67] describe five modifiable elements of audit and feedback that alone produce 288 potential forms of audit and feedback, and Hysong [68] has produced a meta-analysis that documents how different features of audit and feedback impact its effectiveness. Much of what follows regarding specification is intended to further advance better operationalization and contextualization of strategy uses, thereby propelling the field toward a greater understanding of not just what strategies are effective, but how and why they are effective in different contexts [57].

3. **Operationalize it**

Strategies must be described clearly in a manner that ensures that they are discussed at a common level of granularity, are rateable across multiple dimensions, and are readily comparable. In short, they must be defined operationally. This will make implementation strategies more comparable and evaluable, and ultimately make it easier for researchers and other implementation stakeholders to make decisions about which implementation strategies will be most appropriate for their purposes. It will also go a long way toward ensuring that strategies are enacted in the manner intended (*i.e.*, with fidelity). As with clinical interventions, assessing the fidelity of implementation strategy delivery enables a clear test of effectiveness by showing whether or not the strategy was delivered as intended. Without such assessments, it is difficult to determine whether the effectiveness (or lack thereof) of a given strategy can be attributed to the strategy itself or to other contextual factors. An example from another field (human resource management) highlights the utility of carefully operationalizing complex processes. Functional Job Analysis suggests that any given task must include the following information: a) who, b) performs what actions, c) drawing on what knowledge, d) relying on what skills, e) using what materials or tools, f) in order to achieve what outcome? [69].

In a similar fashion, we propose seven dimensions that, if detailed adequately, would constitute the adequate operationalization of implementation strategies: the actors(s)—*i.e.*, who delivers the strategy?; the action(s); the target(s) of the action—*i.e.*, toward what or whom and at what level?; temporality—*i.e.*, when or at what phase?; e) dose—*i.e.*, at what frequency and intensity?; f) the implementation outcome(s) affected; and g) justification—*i.e.*, based upon what theoretical, empirical, or pragmatic justification?. In the following sections, we address each of these dimensions. We provide an illustration of how these dimensions can be specified in Table 2, using two implementation strategies as an example (‘clinical supervision’ and ‘clinician implementation teams’).

a) **The actor**

We define ‘actor’ as a stakeholder who actually delivers the implementation strategy. A wide range of stakeholders can fill this function, as implementation strategies may be employed or enacted by payers, administrators, intervention developers, outside consultants, personnel within an organization charged with being ‘implementers,’ providers/clinicians/support staff, clients/patients/consumers, or community stakeholders. Some strategies could, arguably, be employed only by certain actors. For example, changing reimbursement levels is inherently a ‘payer’ or ‘regulator’ action. Yet other strategies, such as training, could be employed by treatment disseminators external to the organization or supervisors within the organization. Whether certain types of stakeholders are more effective than others in delivering particular strategies is an empirical question; however, there is some theoretical and empirical precedent for relying upon individuals who have more credibility with those whose behavior is expected to change (*e.g.*, the literature on opinion leaders [48,72,73]). Those who report, disseminate, and describe implementation strategies should report details on who enacted the strategy. This will help pave the way for important research on the effect of the ‘actor’ on such outcomes as the strategy’s acceptability to providers, sustainability and implementation costs, and the ultimate effectiveness of the implementation effort.

b) **The action**

Implementation strategies require dynamic verb statements that indicate actions, steps or processes, and sequences of behavior. Ideally, these actions are behaviourally defined a priori to allow comparison with what was actually done during the implementation process. Good examples include strategies such as plan-do-study-act (PDSA) cycles [74] and audit and feedback [66], wherein the very name indicates the actions involved and the definitions expand upon the actions to be taken.

c) **Action target**

The complexity of implementation strategies is also a function of where they are directed or the conceptual ‘targets’ they attempt to impact. For example, strategies such as ‘realigning payment incentives’ target the policy context, while ‘training’ targets front line providers by increasing knowledge and skill, and ‘fidelity checklists’ target the clarity of the intervention as well as the providers’ understanding and ability to break down the intervention into more ‘doable’ steps.

A number of theories, conceptual models, and frameworks point to important ‘targets,’ and most emphasize that implementation strategies may be needed to address multiple ‘targets.’ Rogers’ diffusion of innovation theory, for example, identifies several different targets of implementation efforts related to the innovation itself (*e.g.*, making the EBI more acceptable or seem more ‘doable’), the adopter (*e.g.*, working to make individuals more accepting of innovation), the system adopting the innovation, and the diffusion system [73]. Other models have followed suit in emphasizing the multi-level nature of implementation. For instance, Shortell [75] advances a model with four hierarchical levels involved in any implementation of evidence-based care: the top level, or policy context; two middle levels or organization and group or team; and the bottom level of individual behavior in implementation. The Consolidated Framework for Implementation Research (CFIR) [42], which extends Greenhalgh *et al.*’s [76] seminal model, includes: intervention characteristics (*e.g.*, evidence, adaptability, cost), outer setting (*e.g.*, policies and incentives), inner setting (*e.g.*, structural characteristics of the organization, organizational culture, implementation climate), characteristics of individuals (*e.g.*, self-efficacy), and the process of implementation (*e.g.*, planning, engaging, executing, and reflecting). A recently published checklist for identifying the determinants of practice includes guideline factors; individual health professional factors; patient factors; professional interactions; incentives and resources; capacity for organizational change; and social, political, and legal factors [43]. When the target is an individual, the recently revised Theoretical Domains Framework [44] includes a number of potential targets, such as an individual’s knowledge; skills; roles; optimism; beliefs about consequences; intentions; goals; memory, attention, and decision processes; social influences; emotions; and behavioural regulation. In fact, the multi-level nature of implementation is reflected in the vast majority of pertinent conceptual models. A review of 61 conceptual models pertinent to dissemination and implementation research found that 98% of the included models addressed more than one of the five ‘socioecological levels’ that they specified: system-, community-, organization-, individual-, and policy-levels [41].

Yet too rarely are the specific targets of implementation strategies clearly stated. Specifying the target is necessary because it helps focus the use of the strategy and suggests where and how outcomes should be measured. This is particularly important when reporting complex multifaceted implementation strategies, and the notion here is to be as specific as possible and to rely upon existing conceptual models and frameworks to identify relevant targets.

d) **Temporality**

The order or sequence of strategy use may be critical in some cases. For instance, Lyon *et al.*[77] suggest that strategies to boost providers’ motivation to learn new treatments may need to precede other common implementation strategies such as training and supervision. Several ‘branded’ multifaceted implementation strategies such as ARC organizational implementation strategy [27,28,35], the Replicating Effective Practices framework [32,33], and the Getting to Outcomes framework [31] also lend support to the potential importance of temporality by suggesting specific sequences for the application of component implementation strategies across implementation stages.

The phased nature of implementation is also highlighted in several theories, conceptual models, and frameworks. Fixsen *et al.*[23] suggest six stages of implementation, including exploration and adoption, program installation, initial implementation, full operation, innovation, and sustainability. More recently, Damschroder *et al.*[42] distinguished four processes: planning, engaging, executing, and reflecting/evaluating. In a conceptual model of implementation in public service sectors, Aarons *et al.*[78] also note four phases of implementation, including: exploration, adoption decision, active implementation, and sustainment. Accordingly, implementation strategies may vary in appropriateness and effectiveness across such phases. For example, the strategies needed in the planning stage of implementing interventions may be different from the strategies required to sustain them, once successfully implemented. In their paper on the Dynamic Adaptation Process, Aarons *et al.*[79] illustrate strategy variation across three phases: adoption decision/preparation, active implementation, and sustainment.

Articles that report the use of strategies should include information about the stage or phase when the strategy was used. This should include start and stop dates of strategy use, along with any information about dosage decreasing or increasing over time. Researchers who test strategies need to address the challenges of repeated data collection and analysis. As we come to learn more about the relationships between strategy appropriateness and implementation phases, implications for strategy specification and measurement will become clearer.

e) **Dose**

Just as the intervention or treatment literature addresses the concept of dose, implementation strategies also can vary tremendously in dosage or intensity. Studies of the effectiveness and comparative effectiveness of implementation strategies should measure dose. This is particularly important, because the field needs to know the minimal dose required to get the strongest effect. Thus, details about the dose or intensity of implementation strategies such as the amount of time spent with an external facilitator [39], the time and intensity of training [80], or the frequency of audit and feedback [81] should be designated a priori and reported.

f) **The implementation outcome affected**

Proctor *et al.*[47] proposed a taxonomy of implementation outcomes (acceptability, adoption, appropriateness, feasibility, fidelity, implementation cost, penetration and sustainability). Certain strategies may target one or more these implementation outcomes (or other outcomes not identified in the Proctor *et al.*[47] taxonomy). For instance, using consensus meetings to decide which treatment to implement may be designed to increase the acceptability of the treatment from the perspective of multiple stakeholders. Training or educational strategies typically target fidelity, while financial and policy strategies likely enhance feasibility and acceptability. More information about implementation outcomes can be found in reviews by Proctor *et al.*[47,49,50], and we direct readers to the dissemination and implementation section of the Grid Enabled Measurement Initiative and the Seattle Implementation Research Collaborative’s measures project for repositories of implementation outcomes [82,83]. Researchers or practice leaders who develop, design, and test implementation strategies should explicitly state the implementation outcomes targeted by the strategy.

g) **The justification**

Researchers should make efforts to provide justification or rationale for the strategies that they use to implement a given intervention [57,84]. The selection of implementation strategies may be justified by prospective assessments that identify potential needs, barriers, or facilitators—sometimes termed ‘determinants of practice’ [43,55,85,86]. While these determinants of practice could be identified through formal assessment processes, they could also be identified using theory or conceptual models *e.g.*, [87], research literature *e.g.*, [59,60,88-90], or more informal approaches such as brainstorming *e.g.*, [55]. One these determinants of practice are identified, researchers should attempt to provide clear justification for why the particular strategies were selected (*i.e.*, why would they help in overcoming barriers and/or leveraging facilitators?). Ideally, they should be selected because relevant theory [52,67], empirical evidence [8], and/or some pragmatic rationale (*e.g.*, using a low-cost, low intensity intervention when theory and evidence for more intensive strategies is not compelling) suggest they may be appropriate to address the specific challenges posed by the implementation context. While the role and importance of theory has been debated [51,54,67,91], providing theoretical justification for the selected implementation strategy can highlight the potential mechanisms by which change is expected to occur, ultimately providing greater insight into how and why the strategies might work. A chosen implementation strategy that cannot be justified theoretically, empirically, and/or pragmatically should be carefully reconsidered.

**Table 2:** Specification of two implementation strategies

**Domain**	**Strategy: clinical supervision**	**Strategy: clinician implementation team**
Actor(s)	Clinician who is expert in the clinical innovation and recommended by the treatment developer.	A team of clinicians who are implementing the clinical innovation.
Action(s)	Provides clinical supervision via phone to answer questions, review case implementation, make suggestions, and provide encouragement.	Reflect on the implementation effort, share lessons learned, support learning, and propose changes to be implemented in small cycles of change.
Target(s) of the action	Clinicians newly trained in the innovation.	Clinicians newly trained in the innovation.
	Knowledge about the innovation, skills to use the innovation, optimism that the innovation will be effective, and improved ability to access details about how to use the innovation without prompts.	Knowledge about how to use the innovation in this context, intentions to use the innovation, social influences.
Temporality	Clinical supervision should begin within one week following the end of didactic training.	First meeting should be within two weeks of initial training.
Dose	Once per week for 15 minutes for 12 weeks, plus follow-up booster sessions at 20 and 36 weeks.	Once monthly for one hour for the first six months.
Implementation outcome(s) affected	Uptake of the innovation, penetration among eligible clients/patients, fidelity to the protocol of the clinical innovation.	Uptake of the innovation, penetration among eligible clients/patients, fidelity to the protocol of the clinical innovation, sustainability of the innovation.
Justification	Research that suggests that post-training coaching is more important than quality or type of training received [70].	Cooperative learning theory [71].

### Existing reporting guidelines and suggested extensions

We suggest that journals that routinely publish implementation studies could advance knowledge about strategies by formally adopting reporting guidelines and providing them to authors and reviewers. Applying such guidelines not only to implementation trials but also to articles that focus on the intervention being tested would pushing detail about implementation processes in treatment effectiveness trials and thus accelerate our understanding of strategies. This point is underscored by the call for ‘hybrid trials’ that advance knowledge about both the treatment and the implementation [15].

Several existing guidelines are relevant. For instance, *Implementation Science* and several other journals have embraced the WIDER Recommendations [9,92], which call for authors to provide detailed descriptions of interventions (and implementation strategies) in published papers, clarify assumed change processes and design principles, provide access to manuals and protocols that provide information about the clinical interventions or implementation strategies, and give detailed descriptions of active control conditions. The Standards for Quality Improvement Reporting Excellence (SQUIRE) suggest that authors provide, among other things, a description of the intervention (in this case implementation strategy) and its component parts in sufficient detail so that others could reproduce it, an indication of the main factors that contributed to the choice of the intervention, and initial plans for how the intervention was to be implemented, including the specific steps to be taken and by whom (*i.e.*, the intended roles, qualifications, and training of staff) [93]. The Equator Network [94] is a repository of reporting guidelines (*e.g.*, CONSORT and STROBE) that can provide guidance to specific research designs and methodologies utilized in implementation research. However, there is a need for the development of a suite of reporting guidelines for different types of implementation research [3].

We build upon and extend existing guidelines by recommending two standards as outlined above. First, all studies of implementation should name and define the implementation strategies used. Linguistic harmony in implementation science will be advanced if authors label or describe implementation strategies using terms that already appear in a published review article, a strategy compilation or taxonomy, or another primary research article. If and when unique language is introduced to characterize a strategy, the authors should provide a rationale for the new terminology and should clarify how the new strategy label is similar to or conceptually different from labels already in the literature.

Second, all strategies used should be specified or operationalized. In our view, definition and specification should include each of the seven dimensions outlined above. Ideally, descriptions of implementation strategies should be ‘packaged’ in detailed protocols or manuals describing how a given innovation is to be enacted. These manuals can be considered akin to the kinds of manuals that accompany evidence-based psychotherapies, and could then be published in online supplements and appendices to journal articles.

Adopting these guidelines would address many of the current problems that make it difficult to interpret and use findings from implementation research, such as inconsistent labelling, poor descriptions, and unclear justification for specific implementation strategies [9-11,13]. Specifically, it would facilitate meta-analysis and replication (in both research and practice), and would increase the comparability of implementation strategies by allowing them to be described in similar ways. It would also help to accelerate our understanding of how, why, when, and where they work, and our translation of those findings to real-world improvements in healthcare. We welcome dialogue regarding additional considerations for reporting research on implementation, and acknowledge room for national or international consensus processes that could formalize and extend the guidelines we present here. In the meantime, we hope that these suggestions provide much needed guidance to those endeavouring to advance our understanding of implementation strategies.

## Abbreviations

CFIR: Consolidated framework for implementation research; EBIs: Evidence-based interventions; NIH: National institutes of health; PDSA: Plan-do-study act; SQUIRE: Standards for quality improvement reporting excellence.

## Competing interests

The authors declare that they have no competing interests.

## Authors’ contributions

EKP conceived the idea for this paper and wrote the initial draft. BJP, JCM, and EKP contributed to the conceptualization and writing of subsequent drafts. All authors read and approved the final manuscript.

## Authors’ information

EKP directs the Center for Mental Health Services Research at Washington University in St. Louis (NIMH P30 MH085979), the Dissemination and Implementation Research Core (DIRC) of the Washington University Institute of Clinical and Translational Sciences (NCRR UL1RR024992), the Center for Dissemination and Implementation at the Washington University Institute for Public Health, and the Implementation Research Institute (NIMH R25 MH080916).
